# P-851. Retail Clinic AMS: Reducing Azithromycin and Fluoroquinolones by Over 50%

**DOI:** 10.1093/ofid/ofaf695.1059

**Published:** 2026-01-11

**Authors:** Mickey Hart

**Affiliations:** Froedtert Health, Milwaukee, WI

## Abstract

**Background:**

Up to 90% of human antibacterial use occurs in ambulatory settings. An estimated one-third of outpatient antibacterial prescriptions are unnecessary, and half of necessary prescriptions are suboptimal. The Joint Commission began applying antimicrobial stewardship (AMS) requirements to accredited ambulatory health care organizations in January 2020.

With enthusiastic support from the medical director, we sought to optimize azithromycin and fluoroquinolone prescribing in our retail clinics by implementing quarterly audit and feedback with peer comparison for all retail clinic providers.

**Methods:**

Provider engagement was prioritized during implementation. The ambulatory AMS pharmacist was introduced to the providers by providing live education on a topic of the providers’ choice. A second meeting focused on AMS principles and literature specific to the care setting, emphasizing methods to maintain or improve patient satisfaction. Providers then provided feedback on a draft personalized report.

Initial reports were delivered in late August 2023 and included data for the preceding quarter, with subsequent reports being sent three to six weeks after each quarter’s end. Each one or two page report contains figures comparing the provider’s measures to the average of their de-identified peers’, including prescriptions per 1000 visits and mean duration for overall antibacterials, azithromycin, and fluoroquinolones. Reports incorporate color-coded symbols to highlight specific measures for each provider and include written feedback to reinforce desired behaviors and motivate further improvement. Providers can provide anonymous or identified feedback. Reports are continuously improved.

**Results:**

Compared to the pre-implementation year, azithromycin use decreased by 66.1%, fluoroquinolones by 53.1%, and overall antibacterials by 17.2%.

Positive provider feedback and leadership support have prompted requests for expansion of this model to additional ambulatory care settings, including workforce health clinics.

**Conclusion:**

Audit and feedback with peer comparison was well-received by retail clinic providers and led to meaningful decreases in use of targeted antibacterials within the first year.

**Disclosures:**

All Authors: No reported disclosures

